# Preparation and evaluation of a niosomal delivery system containing *G. mangostana* extract and study of its anti-*Acanthamoeba* activity†

**DOI:** 10.1039/d3na01016c

**Published:** 2024-01-09

**Authors:** Suthinee Sangkana, Komgrit Eawsakul, Tassanee Ongtanasup, Rachasak Boonhok, Watcharapong Mitsuwan, Siriphorn Chimplee, Alok K. Paul, Shanmuga Sundar Saravanabhavan, Tooba Mahboob, Muhammad Nawaz, Maria L. Pereira, Polrat Wilairatana, Christophe Wiart, Veeranoot Nissapatorn

**Affiliations:** a School of Allied Health Sciences, Southeast Asia Water Team (SEA Water Team), World Union for Herbal Drug Discovery (WUHeDD), Research Excellence Center for Innovation and Health Products (RECIHP), Walailak University Nakhon Si Thammarat 80160 Thailand nissapat@gmail.com; b School of Medicine, Walailak University Nakhon Si Thammarat 80160 Thailand; c Department of Medical Technology, School of Allied Health Sciences, Research Excellence Center for Innovation and Health Products (RECIHP), Walailak University Thai Buri Nakhon Si Thammarat 80160 Thailand; d Akkhraratchakumari Veterinary College, Walailak University Nakhon Si Thammarat 80160 Thailand; e School of Pharmacy and Pharmacology, University of Tasmania Hobart TAS 7005 Australia; f Department of Biotechnology, Aarupadai Veedu Institute of Technology, Vinayaka Mission's Research Foundation Paiyanoor Chennai Tamil Nadu 603104 India; g Faculty of Pharmaceutical Sciences, UCSI University Kuala Lumpur 56000 Malaysia; h Department of Nano-Medicine Research, Institute for Research and Medical Consultations, Imam Abdulrahman Bin Faisal University Dammam 34212 Saudi Arabia; i CICECO-Aveiro Institute of Materials, University of Aveiro 3810-193 Aveiro Portugal; j Department of Medical Sciences, University of Aveiro 3810-193 Aveiro Portugal; k Department of Clinical Tropical Medicine, Faculty of Tropical Medicine, Mahidol University Bangkok 10400 Thailand; l Institute for Tropical Biology & Conservation, University Malaysia Sabah Kota Kinabalu 88400 Sabah Malaysia

## Abstract

*Garcinia mangostana* extract (GME) has severe pharmacokinetic deficiencies and is made up of a variety of bioactive components. GME has proven its anti-*Acanthamoeba* effectiveness. In this investigation, a GME-loaded niosome was developed to increase its potential therapeutic efficacy. A GME-loaded niosome was prepared by encapsulation in a mixture of span60, cholesterol, and chloroform by the thin film hydration method. The vesicle size, zeta potential, percentage of entrapment efficiency, and stability of GME-loaded niosomes were investigated. The values for GME-loaded niosome size and zeta potential were 404.23 ± 4.59 and −32.03 ± 0.95, respectively. The delivery system enhanced the anti-*Acanthamoeba* activity, which possessed MIC values of 0.25–4 mg mL^−1^. In addition, the niosomal formulation decreased the toxicity of GME by 16 times. GME-loaded niosome must be stored at 4 °C, as the quantity of remaining GME encapsulated is greater at this temperature than at room temperature. SEM revealed the damage to the cell membrane caused by trophozoites and cysts, which led to dead cells. In light of the above, it was found that GME-loaded niosomes had better anti-*Acanthamoeba* activity. The study suggested that GME-loaded niosomes could be used as an alternative to *Acanthamoeba*'s therapeutic effects.

## Abstract 2

*Garcinia mangostana* extract (GME) has severe pharmacokinetic deficiencies and is made up of a variety of bioactive components.

## Graphical abstract

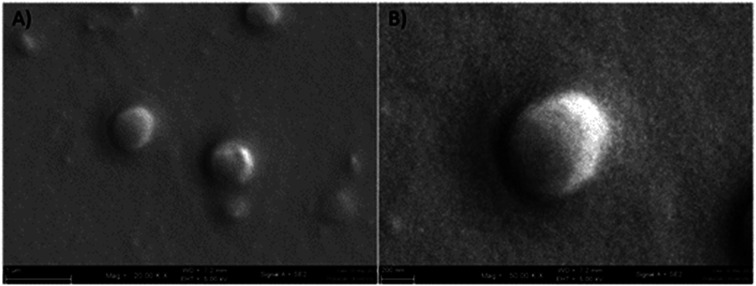

## Introduction

*Garcinia mangostana* is a tropical tree widely distributed in Southeast Asia.^1^ According to reports, *G. mangostana* has a variety of bioactive chemicals with a wide range of biological effects, including those that are anti-oxidant, pro-apoptotic, anti-proliferative, anti-inflammatory, anti-microbial, anti-cancer, and anti-obesity.^2,3^ In our previous study, *G. mangostana* extract (GME), both alone and in combination with chlorhexidine, showed anti-*Acanthamoeba* effectiveness against *A. triangularis* WU19001.^4^ The xanthones that are most prevalent in the pericarp of mangosteen fruit are α-mangostin. Other xanthones found in the mangosteen pericarp include β-mangostin, gartanin, 8-deoxygartanin, garcinones A, B, C, D, and E, mangostinone, 9-hydroxycalabaxanthone, and isomangostin.^5,6^ α-mangostin has a low water solubility (2.03 × 10^4^ mg L^−1^ at 25 °C). In addition, the efficiency of α-mangostin is typically constrained by a first fast metabolism response, an efflux reaction brought on by transporter disruption, fast drug release, and a non-specific target location.^7^ Hence numerous efforts have been undertaken to increase this solubility, including structural modification, co-solvation, solid dispersion, emulsion, complexation, and nanoparticle drug delivery systems.^8–10^

Numerous nanocarrier technologies have been suggested to circumvent the constraint in ocular medicine delivery, including the liposome, niosome, and ethosome. Niosomes are non-ionic surfactant-based vesicles generated by non-ionic surfactants self-assembling in aqueous environments. Researchers in the cosmetics business discovered niosomes in the 1970s, and since then they have frequently been used as drug carriers.^11^ Since this vesicular structure can encapsulate hydrophilic and lipophilic compounds, it is comparable to liposomes (phospholipid vesicles).^12^ Niosomes have various benefits over liposomes, including lower variability in purity, more stability, and improved surfactant availability.^13,14^ Niosomes' non-ionic nature also makes them safe in terms of being genotoxic,^15^ allows for structural flexibility, and enables targeted delivery to a specific area.^16^ Additionally, since niosomes do not need special preparation or storage, they are less costly and more suitable for industrial manufacture than liposomes.^17^

The use of natural product for the treatment of infectious diseases is limited mainly because of low bioavailability. Nanoformulation of extracts and phytochemicals has the potential to enhance their bioavailability, hydrophilicity, stability, and controlled delivery to target sites.^18^ Few nanoformulations have been prepared from chemical constituent of *Garcinia* spp and found to be effective for targeted activities.^18^ A study conducted by Pratiwi revealed the efficacy of self-nanoemulsifying drug delivery system loaded with *G. mangostana* extract against pathogenic bacteria isolated from foot ulcer of diabetic patient.^19^ In a recent study based on wound healing, *G. mangostana* extract was successfully encapsulated in niosome and found to be effectively inhibited the growth of bacteria, cell migration and cell adhesion in an *in vitro* model. Moreover, no toxicity was observed in fibroblast and red blood cells by using this novel drug delivery system.^20^ With the help of nanoparticle drug delivery system, major constituent of GME known as α-mangostin demonstrated enhanced stability, solubility, selectivity and efficacy as an ideal drug candidate.^21^

The purpose of this study was to investigate the preparation of GME-loaded niosome. Moreover, we also evaluated GME-loaded niosome against *Acanthamoeba* species. Furthermore, a scanning electron microscope (SEM) technique was established for the determination and characterization of GME-loaded niosomes on *Acanthamoeba* cells. It is well understood that the *Acanthamoeba* growth-inhibition mechanisms of GME-loaded niosomes provide a safe and efficient way to therapeutically manage *Acanthamoeba* infections.

## Results

### Physicochemical properties

**Fig. 1:**
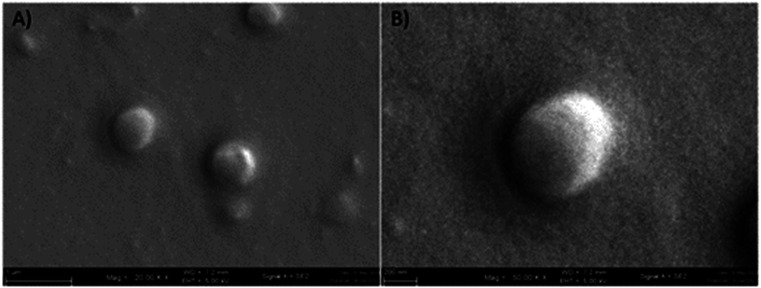
Scanning electron microscope image of GME-loaded niosomes (A), spherical structure of niosome at higher magnification (B).

As a result, a sample generated with a span 60 : cholesterol ratio of 1 : 1 was used to encapsulate GME. Table 1 shows the average hydrodynamic diameter of empty and GME-loaded niosomes. Niosomes loaded with GME are smaller (404.23 ± 4.59 nm) (Fig. 1) than unentrapped niosomes (423.97 ± 16.90 nm). Drug-entrapped niosomes had a zeta potential of −32.03 ± 0.95 mV (Table 1).

**Table 1:** Average size distribution, zeta potential, % yield, % drug loading content (DLC), and % encapsulation efficiency (% EE) of niosomes and GME-loaded niosome

Nanoparticles	Size (nm)	PDI	Charge (mV)	% Yield	% DLC	% EE
Niosomes	423.97 ± 16.90	0.41 ± 0.03	−7.92 ± 0.13	88.93 ± 1.36	n/a	n/a
GME-loaded niosome	404.23 ± 4.59	0.48 ± 0.01	−32.03 ± 0.95	56.87 ± 4.72	18.71 ± 2.17	49.18 ± 7.90

**Fig. 2:**
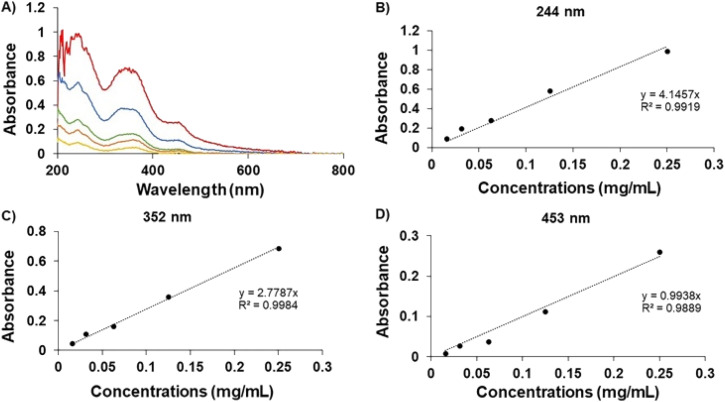
UV-spectrum of GME (A). Standard curve of GME at 244 nm (B), 352NM (C), and 453 nm (D).

In Fig. 2, the UV spectrum demonstrates the peaks of the GME's chromophoric system at 244, 352, and 453 nm. After comparing each standard curve, it can be inferred that the absorbance values of these three wavelengths were employed in linear regression, with the ABS value at 352 nm being proposed since the linear connection (*R*^2^) was the closest to 1. As a result, the amount of GME in the niosome was quantified by dissolving it in ethanol and PBS in a 20 : 80 ratio and measuring it at a wavelength of 352 nm. The data indicated that 49.18 ± 7.90% of GME was entrapped by niosomes (Table 1).

### ATR-FTIR

**Fig. 3:**
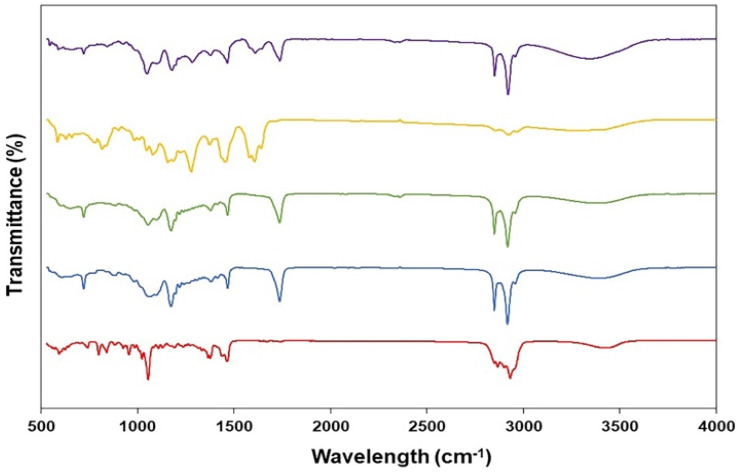
Fourier transformed infrared spectra of cholesterol (red), span60 (blue), niosomes (green), and GME (yellow) along with GME-loaded niosomes (purple).

The ATR-FTIR spectra of pure GME, cholesterol and span 60 are summarised in Fig. 3. The ATR-FTIR spectrum of GME revealed a characteristic peak at 817 cm^−1^ (aldehydes), 1082 cm^−1^ (C–O–C ester) 1278 cm^−1^ (carbon vibration in benzene rings), 1423 cm^−1^ (aromatic compound), 1458 cm^−1^ (C=O vibration), 1606 cm^−1^ (C–C stretching), 1641 cm^−1^ (C=O stretching vibration), 2864 cm^−1^ (C–H group stretching), and 2925 cm^−1^ (Methylene (CH_2_–)). Notably, Fig. 3 (green) illustrates the FTIR spectrum of freeze-dried niosomes powder. All peaks associated with cholesterol and span 60 were present in the niosomes spectra. The cholesterol spectra revealed C–O stretching (1055 cm^−1^), C–H bond stretching (2954 cm^−1^), C–H bond bending (1377 cm^−1^), and –OH stretching (a wide peak in the region of 3000–3700 cm^−1^). C=O stretching (1735 cm^−1^), C–O (1176 cm^−1^), aliphatic CH stretching, asymmetric and symmetric (2916 cm^−1^ and 2850 cm^−1^, respectively), and aliphatic –CH_2_– rocking (721 cm^−1^) were all seen on Span 60. The peaks of GME were seen in a niosome loaded with GME. The lack of peaks in FTIR spectra of GME loaded in niosomes was corroborated by the fact that the GME was loaded in niosomes. Significant hydrogen bonding between formulation components is attributed to a very wide peak of 3000–3700 cm^−1^. According to previous research, the hydrogen bond is more likely to form between GME, cholesterol, and span 60.

### *In vitro* exact release

**Fig. 4:**
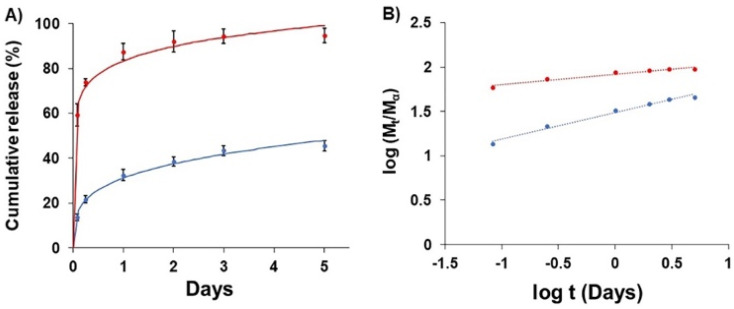
Drug release (A) of free GME (red) and GME-loaded niosomes (blue) and (B) plots of log(*M*_t_/*M*_a_) against log t for GME release from non-carrier (red) and niosome (blue).

The release of GME-loaded niosome (blue line) was compared to that of GME (red line) (Fig. 4), and the quantity of released GME was tracked for up to 5 days. As seen in Fig. 4, encapsulating GME in a niosome may limit burst release. In addition, Fig. 4B shows the release of GME from niosomes through gradual release because the blue line has a higher slope, compared to the release of free GME that could not be controlled, for which the red line has a lower slope.

To explore drug release kinetics in niosomal formulations, release data were analysed using several kinetic models. Several kinetic models were fitted, including quadratic, Baker–Lonsdale, first-order, Hopfenberg, Hixson–Crowell, Higuchi, Korsmeyer–Peppas, and zero-order. In the Korsmeyer–Peppas model (*R*^2^ > 0.99), the following equation explains the release of GME from niosome (Table 2).

**Table 2:** Different kinetic models and their parameters for release of GME from niosomes

Model	Equation	Parameter	*R*_adjusted_^2^
Zero-order	*Q*_R_ = *k*_0_*t*	*k*_0_ = 12.138	−0.7580
First order	*Q*_R_ = 100 (1 − *e*^−*k*_1_*t*^)	*k*_1_ = 0.186	−0.2053
Higuchi	*Q*_R_ = *K*_H_*t*^1/2^	*k*_H_ = 24.604	0.6283
Korsmeyer–Peppas	*Q*_R_ = *k*_KP_*t*^*n*^	*k*_KP_ = 31.135, *n* = 0.268	0.9916
Hixson–Crowell	*Q*_R_ = 100 [1 − (1 − *k*_HC_*t*)^3^]	*k*_HC_ = 0.054	−0.3724
Hopfenberg	*Q*_R_ = 100 [1 − (1 − *k*_HB_*t*)^*n*^]	*k*_HB_ = 0, *n* = 390.233	−0.5082
Baker–Lonsdale	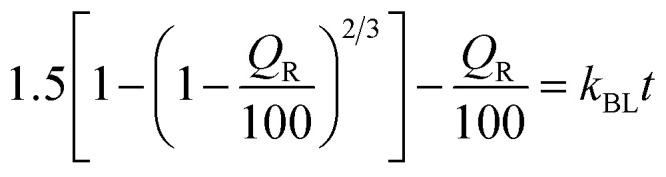	*k*_BL_ = 0.013	0.7372
Quadratic	*Q*_R_ = 100 (*k*_1_*t*^2^ + *k*_2_*t*)	*k*_1_ = −0.040, *k*_2_ = 0.285	0.2743

*M*_t_/*M*_α_ is the proportion of cumulative GME release at a given time, *k* is the constant of drug release rate, and *n* is used to describe release methods such as diffusion or polymer relaxation, and combination mechanisms between diffusion and erosion control. If *n* is smaller than 0.43, diffusion occurs following Fick's law. If *n* is between 0.43 and 0.85, diffusion and relaxation of the niosome influence the release of the GME; however, if *n* is more than 0.85, carrier erosion governs the release. The obtained *n* values (*n* = 0.268) in the Korsmeyer–Peppas model imply that the Fickian diffusion process is responsible for the release of GME from their niosomal formulation.

### Stability test

There was no sedimentation or separation of layers in any of the niosomal dispersion samples. The vesicle diameters of GME-loaded niosomes ranged between 400 and 410 nm. GME loaded with niosomes and dispersed in distilled water exhibited lower vesicular size and fewer negative zeta potential values than blank niosomes. In addition, the presence of the GME may alter the surface charge of the niosomes, as demonstrated in Table 3, hence influencing their size. After 2 and 4 weeks, the GME-loaded niosomes were found to have increased in size. The presence of GME in the niosome structure may have led the niosomes to expand over time, resulting in a growth in size. Moreover, variations in the ambient factor affect temperature. Low temperature, as shown in Table 3, impacts the size of nanoparticles, which tend to become larger due to the increased viscosity of niosomal solution at low temperature. Niosomes are less viscous at room temperature, making them less susceptible to aggregation and fusion, which can result in a more stable size distribution over time. However, higher temperatures induce more drug release than lower temperatures. Consequently, the residual quantity of GME in the niosomes at 4 °C contains a higher percentage of EE and DLC than at room temperature.

**Table 3:** Stability of blank niosome and GME-loaded niosome

Time	Parameters	Blank-niosomes	GME-loaded niosomes
Fresh sample	Size (nm)	423.97 ± 16.90	404.23 ± 4.59
	PDI	0.41 ± 0.03	0.48 ± 0.01
	Charge (mV)	−7.92 ± 0.13	−32.03 ± 0.95
	% yield	88.93 ± 1.36	56.10 ± 3.95
	% DLC	n/a	18.71 ± 2.17
	% EE	n/a	49.18 ± 7.90
2 weeks at 4 °C	Size (mm)	440.33 ± 3.22	479.50 ± 11.82
	PDI	0.42 ± 0.01	0.39 ± 0.04
	Charge (mV)	−40.13 ± 0.57	−30.30 ± 0.26
	% yield	85.38 ± 1.31	53.83 ± 2.97
	% DLC	n/a	17.88 ± 2.12
	% EE	n/a	46.69 ± 7.45
2 weeks at 25 °C	Size (nm)	391.43 ± 6.63	412.43 ± 5.54
	PDI	0.38 ± 0.03	0.40 ± 0.02
	Charge (mV)	−43.07 ± 0.40	−43.13 ± 1.71
	% yield	82.78 ± 1.29	54.70 ± 4.37
	% DLC	n/a	16.88 ± 1.95
	% EE	n/a	44.68 ± 7.37
4 weeks at 4 °C	Size (nm)	385.93 ± 6.42	500.53 ± 9.54
	PDI	0.46 ± 0.02	0.42 ± 0.02
	Charge (mV)	−9.53 ± 1.34	−36.23 ± 1.40
	% yield	80.96 ± 1.24	45.13 ± 2.85
	% DLC	n/a	16.97 ± 4.38
	% EE	n/a	39.51 ± 6.16
4 weeks at 25 °C	Size (nm)	398.13 ± 4.55	462.03 ± 0.51
	PDI	0.39 ± 0.02	0.39 ± 0.01
	Charge (mV)	−2.97 ± 0.26	0.01 ± 0.05
	% yield	81.87 ± 1.31	48.92 ± 3.45
	% DLC	n/a	13.90 ± 1.58
	% EE	n/a	35.28 ± 5.94

### Toxicity

**Fig. 5:**
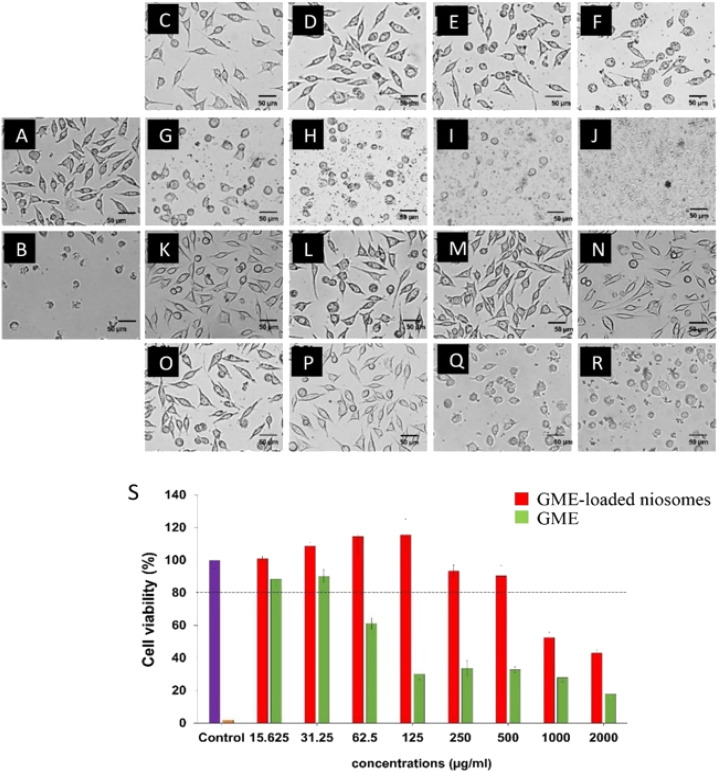
Cytotoxicity of L929 after treated with free GME (C–J) and GME-loaded niosome (K–R). The concentration ranges were between 15.625 and 2000 µg mL^−1^ (S). While, DMEM medium without GME (A) was used as negative control and Triton X-100 (B) was used as positive control.

Cell viability experiments (Fig. 5) demonstrate a decrease in L929 cell viability when GME concentrations increase, as shown by the green bars. GME at a concentration of 31.25 µg mL^−1^ has no cytotoxicity on L929 cells, which have a viability of more than 80% (26). However, GME at 62.5 µg mL^−1^ and above 62.5 µg mL^−1^ concentration may cause cell death. The toxicity is shown to rise with increasing GME concentrations. This demonstrates that increasing the GME concentration has a cytotoxic impact on the L929. This resulted in a decrease in cell viability.

However, it was discovered that niosomes containing GME decreased the cytotoxicity of L929 cells, as shown by the increased survival rate. GME-loaded niosomes of 500 µg mL^−1^ have no cytotoxicity on the L929 cells. This is because niosomes play an essential role in the controlled release of GME. These findings indicated that using niosomes loaded with GME was safer than using GME alone.

### Anti-*Acanthamoeba* activity

The minimum inhibitory concentration (MIC) of GME and GME-loaded niosomes were examined against tested *Acanthamoeba* trophozoites as shown in Table 4. GME showed inhibited activity on *A. castellanii* ATCC30010 at 4 mg mL^−1^ and on *A. triangularis* WU19001 at 0.25 mg mL^−1^, while GME was not active and inhibited *A. castellanii* ATCC50739 and *A. polyphaga* ATCC30461 at 4 mg mL^−1^. The anti-*Acanthamoeba* activities of fresh GME-loaded niosomes were found at 4 mg mL^−1^ against trophozoites and cysts of *A. castellanii* ATCC30010, ATCC50739, and *A. polyphaga* ATCC30461. The MIC values on *A. triangularis* WU19001 were 0.25 and 4 mg mL^−1^ for trophozoites and cysts, respectively. The MIC values of GME-loaded niosome did not change after being stored at 4 °C for 2 and 4 weeks, while the MIC values changed when stored at 25 °C for 4 weeks.

**Table 4:** MIC values of *G. mangostana* extract (GME), GME-loaded niosome against *Acanthamoeba* trophozoites

*Acanthamoeba* strain	MIC value (mg mL^−1^)					
	GME	GME-loaded niosomes				
		Fresh	4 °C	4 °C	25 °C	25 °C
			2 weeks	4 weeks	2 weeks	4 weeks
*A. castellani* ATCC30010	4	4	4	4	4	4
*A. castellani* ATCC50739	>4	4	4	4	4	4
*A. polyphaga* ATCC30461	>4	4	4	4	4	4
*A. triangularis* WU19001	0.25	0.25	0.25	0.25	0.25	0.50

**Fig. 6:**
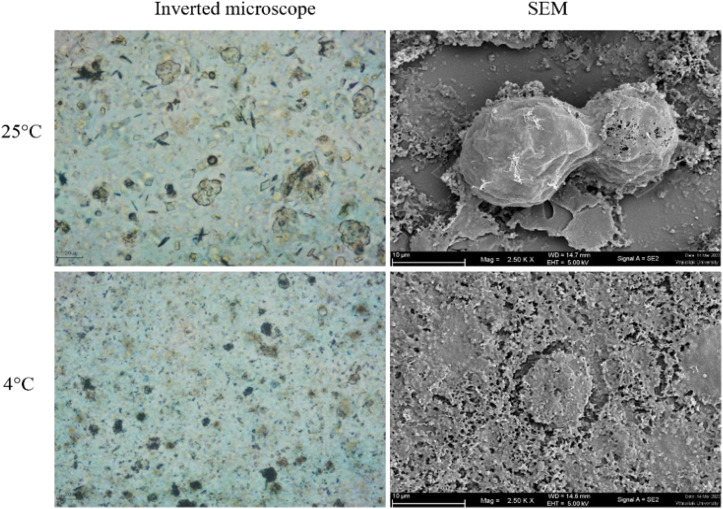
Morphology of *Acanthamoeba triangularis* trophozoites treated by GME-loaded niosomes (250 mg mL^−1^).

**Fig. 7:**
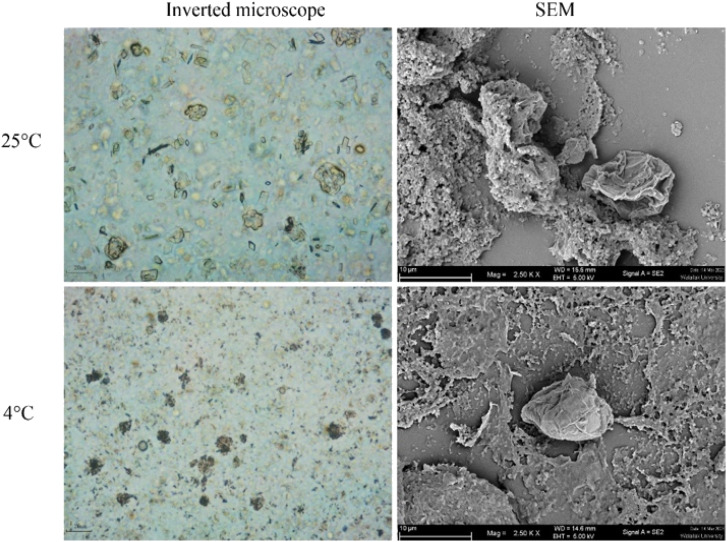
Morphology of *Acanthamoeba triangularis* cysts treated by GME-loaded niosomes (4 mg mL^−1^).

The morphology of *Acanthamoeba* observed by SEM is shown in Fig. 6 and 7. The breakdown of the cell membrane in GME-loaded niosomes was found in both trophozoites and cysts of treated amoeba, which led to the loss of acanthopodia and was found to be the most notable change in the amoeba morphology.

## Discussion

The Mangosteen (*Garcinia mangostana* L.) belongs to the Clusiaceae family that grows in Southeast Asia. Its peels and seeds were used as traditional medicine to treat gastrointestinal and urinary tract infections.^22^ More than 90% of the phytochemical constituents of mangosteen pericarp are xanthones, which are significant polyphenolic compounds, particularly α-mangostin (80–90%) and γ-mangostin (5–10%), also known as panaxanthone.^23^ Obstacles to using mangosteen extract are related to the physicochemical properties of α-mangostin. Its limited therapeutic usefulness is due to its poor water solubility profile and low selectivity for target cells.^21^ Moreover, unsaturated organic acid molecules with short-chain bonds and properties that make them unstable, sensitive, easily reacted with, and oxidized are found in mangosteen peel extract (MPE).^24^ Therefore, nanoparticle formulations could be a helpful technique to resolve such restrictions associated with the therapeutic usefulness of the plant. Among these carriers, niosomes are among the best. Niosomes are microscopic lamellar structures synthesized when cholesterol is combined with a non-ionic surfactant of the alkyl or dialkyl polyglycerol ether family.^25^ A previous publication^26^ proposes combining span 60 with cholesterol in a 1 : 1 ratio to get the maximum amount of drug encapsulation. Chin *et al.*, successfully synthesized α-mangostin niosome to increase the skin permeability of α-mangostin.^21^ Findings from Xu *et al.*, 2017, reported increased bioavailability and absorption in gastrointestinal tract of α-mangostin loaded self-microemulsion.^27^ In another study, the efficacy of water-soluble β-cyclodextrin-coated α-mangostin polymeric nanoparticles on lung cancer cells was enhanced by 2.1-fold as compared to free α-mangostin.^28^ Sakpakdeejaroen and colleagues reported enhanced cellular accumulation when α-mangostin was entrapped in transferrin-conjugated lipid polymer hybrid nanoparticles and found to be effective in antiproliferative activity of various cancer cell lines.^29^ Therefore, transferrin-conjugated lipid polymer hybrid nanoparticle is a highly promising therapeutic system that should be further optimized as therapeutic tools for cancer treatment.^29^

This study we selected a 1 : 1 ratio of span 60 and cholesterol for niosome preparation which it is often based on a combination of factors related to the stability, structural properties, and performance of niosomes. Cholesterol is often added to niosome formulations to improve their stability. Cholesterol can insert itself into the lipid bilayers of niosomes, reducing the fluidity of the bilayers and preventing leakage or fusion of niosomes. A 1 : 1 ratio can provide a balanced composition that enhances the stability of the niosomes by maintaining appropriate bilayer rigidity. Additionally, the 1 : 1 ratio may lead to niosomes with more uniform and consistent particle sizes, which can be advantageous for applications that require precise control over niosome size distribution.^30–33^

Our study used widely distributed GME-loaded niosomes with noticeably smaller particle sizes at 404.23 ± 4.59 nm. However, niosome sizes depending on the production process, the kind of vesicles, the type of surfactant, and the ratio of surfactant to cholesterol. Niosome types used in numerous ocular applications include SUV, LUV, and MLV, which are nano-(10–100 nm), micron- (100–3000 nm) and multilamellar (>5 µm) sized vesicles.^34^ Niosome size should be sufficient for topical applications to prevent removal with tear turnover and drainage. In study of Fitzgerald and collages showed that MLV had a slower clearance rate and a longer precorneal retention period than SUV.^35,36^ Meanwhile, Barza *et al.* demonstrated that for intravitreal application, LUV having a diameter of 600 nm had a longer clearing time from the vitreous humor than SUV with a diameter of 60 nm.^37^ In this study, the size of niosomes was obtained at 404.23 ± 4.59 nm, which is expected to be suitable for future work *in vivo* study.

Interestingly, GME-loaded niosomes have a higher negative zeta potential, indicating the nanoformulation's colloidal stability.^38^ The higher negative zeta potential of GME-loaded niosomes may be caused by ionization of the GME. GME contains functional groups that can ionize in solution, meaning they can either release or take up ions (protons) depending on the pH of the surrounding environment. GME has ionizable groups and the pH of the solution favors their ionization, this can result in a change in the overall charge of the nanoparticles. GME contains acidic groups and becomes ionized, it can introduce negatively charged ions to the nanoparticle surface. Adsorption of Ions is the second reason. When GME are loaded onto niosome, they can bring along ions from the surrounding solution. Some of these ions may be negatively charged, and their adsorption onto the nanoparticle surface can increase the overall negative charge of the nanoparticles. Third, conformational changes, the presence of the GME molecules on the nanoparticle surface may induce conformational changes or reorganization of the surface molecules, including any stabilizing agents or ligands used in the nanoparticle formulation. These changes can expose more negatively charged functional groups on the nanoparticle surface, leading to a more negative surface charge. Lastly, electrostatic interactions, depending on the charge of GME and the surface properties of the nanoparticles, electrostatic interactions can come into play. GME has a net negative charge, it may electrostatically repel other negatively charged groups on the nanoparticle surface, causing them to become more exposed and contributing to a more negative charge.^39–42^Fig. 2D showed that the GME-loaded niosomes were stable at room temperature on the day of production. Niosome stability is significantly influenced by the size and charge of the niosomes. According to Ag Seleci *et al.* (2016),^43^ the optimal size of the niosomes ranges from 10 to 3000 nm. However, niosomes must be ≤1000 nm for the successful encapsulation of drugs, good stability, and better release kinetics.^44^ It is noteworthy in this study, the charge of GME-loaded niosomes becomes nearly neutral when stored at 25 °C for 4 weeks. This may be caused by surfactant degradation. Surfactant can undergo degradation when exposed to 25 °C. High temperatures can lead to the breakdown of the surfactant molecules, resulting in a loss of their amphiphilic properties. This degradation can disrupt the structure of the niosomes and affect their surface charge, potentially neutralizing it. Another possible reason is about vesicle fusion. Elevated temperatures can increase the kinetic energy of the molecules in the niosomal system, leading to increased molecular motion and collision frequency. This can promote the fusion or coalescence of individual niosomes, causing them to merge and form larger vesicles. As niosomes fuse, their surface area increases, potentially diluting the charge, making it less pronounced, and even neutralizing it in some cases.^45,46^ Particle aggregation is less likely to develop in niosomes with a high surface potential, owing to their greater repulsion potential, which enables them to maintain their long-term stability. Span60 was used in the formulation of niosomes because its chemical structure is appropriate. Span 60 has a smaller head group (sorbitan) as compared to Tween 60 (polyoxyethylene), which decreased the fluidity and permeability of the bilayer, which in turn improved the drug entrapment.^47^ Formation of hydrogen bonds between the 3 beta-hydroxy groups of the cholesterol and the oxygen at the sorbitan monostearate ester group produces in highly structured gel formation, leading to less leakage and higher EE.^48^ According to the FTIR results, the possibility of hydrogen bonding between GME, cholesterol, and span 60 occur and have a positive impact on %EE. GME-loaded niosomes, besides their ability to save the GME from expected biodegradation in the body, also promoted the sustained release of GME over 5 days (Fig. 4). It is possible to think of the release profile for GME-loaded niosomes as a two-step procedure. Day 1 started with an initial burst release that might have been caused by GME diffusing from its surface-adsorbed position. The diffusion of GME into the inner layers of the niosomes may have contributed to the second stage's slower release (late stage of day 1 to day 5).^49,50^ In addition, nanonized particle drug delivery systems are becoming more popular because of their clear benefits, including decreased dosage, decreased toxicity and dose-related adverse effects, and increased patient compliance.^51^ This study discovered that GME-loaded niosomes decreased the toxicity of L929 cells when compared with free GME. Because the bioactive agent's encapsulation in niosomes inhibits drug inactivation, directs the biologically active substance to the target tissue, and enables delayed release of the loaded medication into the circulatory system, so minimizing its toxicity.^52^

GME-loaded niosomes were effective at 0.25 on *A. triangularis* WU19001 and 4 mg mL^−1^ on all strains of the same type of *Acanthamoeba*, while free GME was effective on two strains, *A. castellanii* ATCC30010 and *A. triangularis* WU19001. This might be because the niosomes, which are surfactant vesicles, enhance GME entry into the cells and can improve the solubility and stability of GME. There are few antimicrobial medications that can effectively treat *Acanthamoeba* infections in people. Chlorhexidine, along with diamidines and neomycin, is the current treatment for *Acanthamoeba* keratitis. It can last up to a year, although even then, about 10% of patients report infection recurrence.^53^ In addition, most medications have a high human toxicity level and can lead to unfavourable side effects. Thus, finding substances that enhance the antiparasitic action of medicines on *Acanthamoeba* is crucial and helpful. According to our previous results, free GME showed synergistic interaction in the anti-*Acanthamoeba* activity of chlorhexidine that can be used against the cyst of *A. triangularis* (WU19001). After being stored at 4 °C for 4 weeks, the charge of niosomes has a higher SD because the size of the niosomes has varied greatly, resulting in the precipitation of larger niosomes. These size and charge characteristics were consistent with the drug loading results.

## Methods

### Preparation of plant extracts

*G. mangostana* extract (GME) was prepared from a previous study.^4^ The dry pericarp of *G. mangostana* Linn. was purchased from an herbal shop. The 50 g of dry powder was soaked in 200 mL of ethanol for 7 days and filtered through Whatman No. 1 (GE Healthcare Life Science, Buckinghamshire HP79NA, United Kingdom). The supernatant was evaporated to dryness under reduced pressure using a rotary evaporator to obtain *G. mangostana* extract (GME).

### Preparation of GME-loaded niosomes

Niosomes were synthesised utilizing the thin-film hydration approach using span 60 (a non-ionic surfactant) and cholesterol.^54^ To begin, span 60 and cholesterol were dissolved in chloroform at a ratio of 1 : 1 M.^26^ Then, 10 mg of GME was added to the solution as a model of anti-*Acanthamoeba*.^4^ To achieve the thin layer on a container, the liquid was agitated for 15 minutes before the chloroform was evaporated. For 30 minutes, the film was hydrated with 10 mL distilled (DI) water and swirled. The fabricated niosomes were sonicated for two minutes at an amplitude of 80% using a probe sonicator and maintained at 4 °C. For subsequent studies, the materials were stored in a refrigerator (4 °C).

### Particle size and charge of GME-loaded niosomes

After dispersing niosomes in deionised water, the vesicle size and charge of the GME-loaded niosome formulation were determined using the dynamic light scattering (DLS) method and a Malvern Zeta Sizer (Malvern Instruments, UK).^55^ Each sample was measured in three repetitions at a temperature of 25 °C and a laser wavelength of 633 nm, with a scattering angle of 173°, a medium viscosity of 0.8872 cP, and a refractive index of 1.33.

### Determination of yield, encapsulation efficiency, and drug loading content

The yield, encapsulation efficiency (EE), and drug loading content (DLC) of niosomes were determined.^56^ First, the free drug was extracted from niosomes using centrifugation at 18 000 rpm for 35 minutes at 4 °C. The pellet was suspended in DI water and then freeze-dried to get the powder. The powder was then weighed, the yield was estimated (eqn (1)) and it was dissolved in a 20 : 80 mixture of ethanol and PBS to certify the encapsulated GME. The extracted solution was quantified for drug concentration, and then the drug loading content and EE were computed (eqn (2) and (3)). Triplicate trials were conducted.

1 % yield = (weight of nanoparticle)/(theoretical total amount of nanoparticle) × 100

2 % DLC = (amount of encapsulated GME in niosome)/(weight of nanoparticle) × 100

3 % EE = (amount of encapsulated GME in niosome)/(initial amount of GME) × 100

### ATR-FTIR examination

To determine the GME–excipient interaction, an FT-IR spectrophotometer (Bruker Tensor 27 series, Germany) equipped with a diamond ATR (attenuated total reflectance) was employed. The ATR-FTIR analysis was performed on niosomes, GME, and GME-loaded niosomes powder (freeze-dried formulation). The spectra were obtained by pouring a small quantity of each homogenous sample onto the window and scanning between wavenumbers 400–4000 cm^−1^ with a resolution of 2 cm^−1^.

### *In vitro* release study of GME-loaded niosomes

Before observing the release profile of GME-loaded niosomes, niosomes were centrifuged to separate them from the free drug. The niosomes containing GME were then transferred to a dialysis bag with a molecular weight cutoff of 50 kDa (MWCO, Spectrum Labs, USA). In an incubator shaker, the bag was submerged in phosphate buffer saline (PBS, pH 7.4) and incubated at 37 °C and 90 rpm. Fifteen mL of solution was changed and replaced with 15 mL of fresh PBS at predetermined time intervals.^57^ Additionally, free GME was examined. The collected material was diluted with ethanol (20% v/v), and the drug content was evaluated by NanoDrop spectroscopy.

### Stability test

Niosomal dispersions were deposited in 20 mL glass vials and kept both at room temperature and at 4 °C in the refrigerator to evaluate the stability of niosome formulations. After preparation, the size, PDI, and zeta potential of the formulations were evaluated at certain time intervals (0, 2, and 4 weeks). In addition, the amount of GME remaining in the niosomes was determined, including measures of % yield, % DLC, and %EE.

### *In vitro* cytotoxic test

Samples were evaluated for cytotoxicity in L929 murine fibroblast cells using ISO 10993-5. The MTT test was used to examine the test samples, the negative control (culture medium), and the positive control (1% H_2_O_2_) in triplicate.^56^ 96-well plates were seeded with L929 at a density of 5 × 10^3^ cells per 100 µL medium. After 24 hours of incubation, the cells were washed with a medium, and the various GME and GME-loaded niosomes concentrations were added. After treatment, the MTT test was used to assess cell viability. The blue-purple colour shift was achieved by dissolving formazan in DMSO and measured at a 570 nm microplate reader (TECAN). The results were given as mean ± standard deviation (SD).

### Organisms

The four strains of *Acanthamoeba*, namely *A. triangularis* WU19001, an environmental strain from Walailak University, *A. castellanii* ATCC50739, *A. castellanii* ATCC30010, and *A. polyphaga* ATCC30461 from the American Type Culture Collection, were used in this study. The parasite was grown in Peptone-Yeast-Glucose Medium (PYG) [20 g proteose peptone, 2 g yeast extract, 0.98 g MgSO_4_·7H_2_O, 0.35 g Na_2_HPO_4_·7H_2_O, 0.34 g KH_2_PO_4_, 0.02 g (NH_4_)_2_Fe(SO_4_)_2_·6H_2_O, 18 g glucose, distilled water, 1 L]. The trophozoites were observed after 72 hours of incubation at room temperature in a dark condition. Then, the parasites were centrifuged at 3000 rpm for 5 minutes and re-suspended in fresh PYG. The 90% mature cysts were obtained from Neff's medium. The viability was investigated using a 0.2% trypan blue assay and adjusted to 2 × 10^5^ cells per mL using a hemocytometer (Boeco, Hamburg, Germany) before used.^58^

### Anti-*Acanthamoeba* activity

A broth microdilution assay was performed to determine the minimum inhibitory concentration for GME, GME-loaded niosomes, and blank niosomes against four strains of *Acanthamoeba* trophozoites and cysts. The GME-loaded niosomes were diluted to give a final concentration of 4, 2, 1, 0.5, 0.25, 0.125 mg mL^−1^ in a 96-well microplate. Then 100 µL of 2 × 10^5^ cells per mL of trophozoites and cysts were inoculated into each well. Then the plates were incubated at 28 °C for 24 hours. The cell viability was analyzed under the inverted microscope by trypan blue staining. For counting, 50 µL of cell suspension was mixed with 50 µL of 0.2% trypan blue. Viability was counted with a hemacytometer (Boeco, Hamburg, Germany) under the inverted microscope based on the principle of the dye can cross the membrane of dead cells with blue color, but not intact membrane of viable cells with colorless appearances. All experiments were performed according to the biosafety guidelines for scientific research at Walailak University, Nakhon Si Thammarat, Thailand (ref. no. WU-IBC-66-020).

### Scanning electron microscopic (SEM) study

The effects of individual GME-loaded niosomes on *A. triangularis* trophozoites and cysts were determined by a SEM study. *Acanthamoeba* trophozoites and cysts were treated with MIC concentrations of GME-loaded niosomes (0.25 and 4 mg mL^−1^). After incubation, cells were collected by centrifugation at 3000 rpm for 5 minutes and re-suspended in phosphate buffer saline (PBS). Cells in 1% DMSO were used as negative controls. Samples were fixed with 2.5% glutaraldehyde overnight. The samples were further dehydrated with a series of graded alcohol (20%, 40%, 60%, 80%, 90%, and 100% ethanol), mounted on aluminum stubs, and allowed to dry using a critical point dryer, EMS/K850 (Quorum, Laughton, UK). Samples were then coated with gold particles, and the morphology of *Acanthamoeba* trophozoites after treatment was subsequently examined under SEM (SEM-Zeiss, Munich, Germany) at the center for Scientific and Technological Equipment, Walailak University, Nakhon Si Thammarat, Thailand.

## Author contributions

S. S., K. E. and V. N. conceptualized the experiments, S. S., K. E. and T. O. devised methodology, performed validation and conducted formal analysis, S. S., K. E. and T. O. prepared the original draft, R. B., W. M., A. K. P., S. S. S., T. M., M. N., M. L. P., P. W., S. C., M. R. and C. W. finalized the manuscript. All authors reviewed the manuscript.

## Conflicts of interest

There are no conflicts of interest to declare.

## Supplementary Material

## References

[cit1] Taher M., Tg Zakaria T., Susanti D., Zakaria Z. A. (2016). Hypoglycaemic activity of ethanolic extract of *Garcinia mangostana* Linn. in normoglycaemic and streptozotocin-induced diabetic rats. BMC Complementary Altern. Med..

[cit2] Janardhanan S., Mahendra J., Girija A. S., Mahendra L., Priyadharsini V. (2017). Antimicrobial effects of *Garcinia mangostana* on cariogenic microorganisms. J. Clin. Diagn. Res..

[cit3] Ovalle-Magallanes B., Eugenio-Pérez D., Pedraza-Chaverri J. (2017). Medicinal properties of mangosteen (*Garcinia mangostana* L.): a comprehensive update. Food Chem. Toxicol..

[cit4] Sangkanu S., Mitsuwan W., Mahabusarakam W., Jimoh T. O., Wilairatana P., Girol A. P., Verma A. K., de Lourdes Pereira M., Rahmatullah M., Wiart C., Siyadatpanah A., Norouzi R., Mutombo P. N., Nissapatorn V. (2021). Anti-*Acanthamoeba* synergistic effect of chlorhexidine and *Garcinia mangostana* extract or α-mangostin against *Acanthamoeba triangularis* trophozoite and cyst forms. Sci. Rep..

[cit5] Nauman M. C., Johnson J. J. (2022). The purple mangosteen (*Garcinia mangostana*): defining the anticancer potential of selected xanthones. Pharmacol. Res..

[cit6] Ovalle-Magallanes B., Eugenio-Pérez D., Pedraza-Chaverri J. (2017). Medicinal properties of mangosteen (*Garcinia mangostana* L.): a comprehensive update. Food Chem. Toxicol..

[cit7] Li L., Brunner I., Han A., Hamburger M., Kinghorn A. D. (2011). Pharmacokinetics of a -mangostin in rats after intravenous and oral application. Mol. Nutr. Food Res..

[cit8] Rungnim C., Phunpee S., Kunaseth M., Namuangruk S., Rungsardthong K., Rungrotmongkol T., Ruktanonchai U. (2015). Co-solvation effect on the binding mode of the α-mangostin/β-cyclodextrin inclusion complex. Beilstein J. Org. Chem..

[cit9] Zarena A. S., Sankar K. U. (2015). Synthesis of α− mangostin-D-glucoside in supercritical carbon dioxide media. J. Food Sci. Technol..

[cit10] Elsaid Ali A. A., Taher M., Mohamed F. (2013). Microencapsulation of alpha-mangostin into PLGA microspheres and optimization using response surface methodology intended for pulmonary delivery. J. Microencapsulation.

[cit11] Uchegbu I. F., Vyas S. P. (1998). Non-ionic surfactant based vesicles (niosomes) in drug delivery. Int. J. Pharm..

[cit12] Yoshioka T., Sternberg B., Florence A. T. (1994). Preparation and properties of vesicles (niosomes) of sorbitan monoesters (Span 20, 40, 60 and 80) and a sorbitan triester (Span 85). Int. J. Pharm..

[cit13] Rahimpour Y., Hamishehkar H. (2012). Niosomes as carrier in dermal drug delivery. Recent Adv. Novel Drug Carrier Syst..

[cit14] Kapil S., Rao R., Saini V. (2012). Preparation and evaluation of lornoxicamniosomal gel. Int. Res. J. Pharm..

[cit15] Marianecci C., Di Marzio L., Rinaldi F., Celia C., Paolino D., Alhaique F., Esposito S., Carafa M. (2014). Niosomes from 80s to present: the state of the art. Adv. Colloid Interface Sci..

[cit16] Chen S., Hanning S., Falconer J., Locke M., Wen J. (2019). Recent advances in non-ionic surfactant vesicles (niosomes): fabrication, characterization, pharmaceutical and cosmetic applications. Eur. J. Pharm. Biopharm..

[cit17] Verma A. K., Bindal M. (2012). A review on niosomes: an ultimate controlled and novel drug delivery carrier PAPER WITHDRAWN. Int. J. Nanomed..

[cit18] Baky H. M., Fahmy H., Farag M. A. (2022). Recent advances in *Garcinia cambogia* nutraceuticals in relation to its hydroxy citric acid level. A comprehensive review of its bioactive production, formulation, and analysis with future perspectives. ACS Omega.

[cit19] Pratiwi L. (2021). Antibacterial Activity of Self-Nanoemulsifying Drug Delivery System (SNEDDSS) Loaded with Mangosteen (*Garcinia mangostana* L.,) Peels against *Bacilus subtitis, Bacillus cereus*, and *Staphylococcus aureus* isolated from diabetic foot ulcer patients. Majalah Obat Tradisional.

[cit20] Pooprommin P., Manaspon C., Dwivedi A., Mazumder A., Sangkaew S., Wanmasae S., Tangpong J., Ongtanasup T., Eawsakul K. (2022). Alginate/pectin dressing with niosomal mangosteen extract for enhanced wound healing: evaluating skin irritation by structure-activity relationship. Heliyon.

[cit21] Wathoni N., Rusdin A., Motoyama K., Joni I. M., Lesmana R., Muchtaridi M. (2020). Nanoparticle drug delivery systems for α-mangostin. Nanotechnol., Sci. Appl..

[cit22] Kosem N., Ichikawa K., Utsumi H., Moongkarndi P. (2013). In *vivo* toxicity and antitumor activity of mangosteen extract. J. Nat. Med..

[cit23] Matsumoto K., Akao Y., Kobayashi E., Ohguchi K., Ito T., Tanaka T., Iinuma M., Nozawa Y. (2003). Induction of apoptosis by xanthones from mangosteen in human leukemia cell lines. J. Nat. Prod..

[cit24] Boots A. W., Haenen G. R., Bast A. (2008). Health effects of quercetin: from antioxidant to nutraceutical. Eur. J. Pharmacol..

[cit25] Malhotra M., Jain N. K. (1994). Niosomes as drug carriers. Indian Drugs.

[cit26] Ghafelehbashi R., Akbarzadeh I., Yaraki M. T., Lajevardi A., Fatemizadeh M., Saremi L. H. (2019). Preparation, physicochemical properties, *in vitro* evaluation and release behavior of cephalexin-loaded niosomes. Int. J. Pharm..

[cit27] Xu W. K., Jiang H., Yang K., Wang Y. Q., Zhang Q., Zuo J. (2017). Development and *in vivo* evaluation of self-microemulsion as delivery system for α-mangostin. Kaohsiung J. Med. Sci..

[cit28] Phuong N. T. M., Dai Lam T., Mai T. T., Hop N. T. (2018). Cytotoxicity of α-mangostin encapsulated polymeric nanoparticles against lung cancer cells. Acad. J. Biol..

[cit29] Sakpakdeejaroen I., Muanrit P., Panthong S., Ruangnoo S. (2022). Alpha-mangostin-loaded transferrin-conjugated lipid-polymer hybrid nanoparticles: development and characterization for tumor-targeted delivery. Sci. World J..

[cit30] Balakrishnan P., Shanmugam S., Lee W. S., Lee W. M., Kim J. O., Oh D. H., Yong C. S. (2009). Formulation and *in vitro* assessment of minoxidil niosomes for enhanced skin delivery. Int. J. Pharm..

[cit31] Pando D., Gutiérrez G., Coca J., Pazos C. (2013). Preparation and characterization of niosomes containing resveratrol. J. Food Eng..

[cit32] Bhaskaran S., Lakshmi P. K. (2009). Comparative evaluation of niosome formulations prepared by different techniques. Acta Pharm. Sci..

[cit33] Auda S. H., Fathalla D., Fetih G., El-Badry M., Shakeel F. (2016). Niosomes as transdermal drug delivery system for celecoxib: *in vitro* and *in vivo* studies. Polym. Bull..

[cit34] Gharbavi M., Amani J., Kheiri-Manjili H., Danafar H., Sharafi A. (2018). Niosome: a promising nanocarrier for natural drug delivery through blood–brain barrier. Adv. Pharmacol. Sci..

[cit35] Fitzgerald P., Hadgraft J., Kreuter J., Wilson C. G. (1987). A γ-scintigraphic evaluation of microparticulate ophthalmic delivery systems: liposomes and nanoparticles. Int. J. Pharm..

[cit36] Fitzgerald P., Hadgraft J., Wilson C. G. (1987). A gamma scintigraphic evaluation of the precorneal residence of liposomal formulations in the rabbit. J. Pharm. Pharmacol..

[cit37] Barza M., Stuart M., Szoka F. (1987). Effect of size and lipid composition on the pharmacokinetics of intravitreal liposomes. Invest. Ophthalmol. Visual Sci..

[cit38] Midekessa G., Godakumara K., Ord J., Viil J., Lättekivi F., Dissanayake K., Kopanchuk S., Rinken A., Andronowska A., Bhattacharjee S., Rinken T., Fazeli A. (2020). Zeta potential of extracellular vesicles: toward understanding the attributes that determine colloidal stability. ACS Omega.

[cit39] Saraswati D. H., Harumi M., Triyono T., Sudiono S. (2020). The effectiveness adsorption of Au (III) and Cu (II) ion by mangosteen rind (*Garcinia mangostana* L.) using point of zero charge calculation. Key Eng. Mater..

[cit40] Manosroi A., Khanrin P., Lohcharoenkal W., Werner R. G., Götz F., Manosroi W., Manosroi J. (2010). Transdermal absorption enhancement through rat skin of gallidermin loaded in niosomes. Int. J. Pharm..

[cit41] Simón-Vázquez R., Lozano-Fernández T., Peleteiro-Olmedo M., González-Fernández Á. (2014). Conformational changes in human plasma proteins induced by metal oxide nanoparticles. Colloids Surf., B.

[cit42] Forest V., Cottier M., Pourchez J. (2015). Electrostatic interactions favor the binding of positive nanoparticles on cells: a reductive theory. Nano Today.

[cit43] García-Manrique P., Machado N. D., Fernández M. A., Blanco-López M. C., Matos M., Gutiérrez G. (2020). Effect of drug molecular weight on niosomes size and encapsulation efficiency. Colloids Surf., B.

[cit44] Ag Seleci D., Seleci M., Walter J.-G., Stahl F., Scheper T. (2016). Niosomes as nanoparticular drug carriers: fundamentals and recent applications. J. Nanomater..

[cit45] Holmberg K. (2001). Natural surfactants. Curr. Opin. Colloid Interface Sci..

[cit46] Markowitz M. A., Dunn D. N., Chow G. M., Zhang J. (1999). The effect of membrane charge on gold nanoparticle synthesis via surfactant membranes. J. Colloid Interface Sci..

[cit47] Bayindir Z. S., Yuksel N. (2010). Characterization of niosomes prepared with various nonionic surfactants for paclitaxel oral delivery. J. Pharm. Sci..

[cit48] Nasseri B. (2005). Effect of cholesterol and temperature on the elastic properties of niosomal membranes. Int. J. Pharm..

[cit49] Hosseini S. F., Zandi M., Rezaei M., Farahmand F. (2013). Two-step method for encapsulation of oregano essential oil in chitosan nanoparticles: preparation, characterization and *in vitro* release study. Carbohydr. Polym..

[cit50] Keawchaoon L., Yoksan R. (2011). Preparation, characterization and *in vitro* release study of carvacrol-loaded chitosan nanoparticles. Colloids Surf., B.

[cit51] Sahin N. O. , Niosomes as nanocarrier systems, in Nanomaterials and Nanosystems for Biomedical Applications, Springer, 1st edn, 2007, pp. 67–81

[cit52] Siddiqui R., Aqeel Y., Khan N. A. (2016). The development of drugs against *Acanthamoeba* infections. Antimicrob. Agents Chemother..

[cit53] Derda M., Hadaś E., Thiem B. (2009). Plant extracts as natural amoebicidal agents. Parasitol. Res..

[cit54] Dwivedi A., Mazumder A., Nasongkla N. (2018). Layer-by-layer nanocoating of antibacterial niosome on orthopedic implant. Int. J. Pharm..

[cit55] De Silva L., Fu J.-Y., Htar T. T., Muniyandy S., Kasbollah A., Kamal W. H. B., Chuah L. H. (2019). Characterization, optimization, and *in vitro* evaluation of Technetium-99m-labeled niosomes. Int. J. Nanomed..

[cit56] Nasongkla N., Tuchinda P., Munyoo B., Eawsakul K. (2021). Preparation and characterization of MUC-30-loaded polymeric micelles against MCF-7 cell lines using molecular docking methods and *in vitro* study. Evidence-Based Complementary Altern. Med..

[cit57] Eawsakul K., Tancharoen S., Nasongkla N. (2021). Combination of dip coating of BMP-2 and spray coating of PLGA on dental implants for osseointegration. J. Drug Delivery Sci. Technol..

[cit58] Mitsuwan W., Sin C., Keo S., Sangkanu S., de Lourdes Pereira M., Jimoh T. O., Salibay C. C., Nawaz M., Norouzi R., Siyadatpanah A., Wiart C. (2021). Potential anti-*Acanthamoeba* and anti-adhesion activities of *Annona muricata* and *Combretum trifoliatum* extracts and their synergistic effects in combination with chlorhexidine against *Acanthamoeba triangularis* trophozoites and cysts. Heliyon.

